# Progressing participatory research with young people in India: how a youth advisory board supported a large mental health project in Bengaluru

**DOI:** 10.1186/s40900-025-00781-5

**Published:** 2025-10-14

**Authors:** Mutharaju Arelingaiah, Janardhan Navaneetham, Krupa Arasanahalli Lakshman, Jayalaxmi Kaniyagundi Podiya, Sphoorti G. Prabhu, Prachi Kandeparker, Ritwika Nag, Siobhan Hugh-Jones

**Affiliations:** 1 Department of Psychiatric Social Work, National Institute of Mental Health and Neurosciences, Bengaluru, India; 2 Department of Psychiatric Social Work, National Institute of Mental Health and Neurosciences, Bengaluru, India; 3 Department of Psychiatric Social Work, National Institute of Mental Health and Neurosciences, Bengaluru, India; 4 Sangath, Goa, India; 5 School of Psychology, University of Leeds, Leeds, UK

**Keywords:** Patient and public involvement and engagement, Youth advisory boards, Adolescent mental health, India, Whole school programs

## Abstract

**Background:**

Action is needed to advance youth mental health research in India, and young people could significantly contribute to this. However, youth involvement in Indian research is rare, largely due to cultural norms around how research is done and the place of young people in society. There are calls for guidance on how youth involvement could be accelerated in India in ways that respect cultural norms. Youth Advisory Boards (YABs) are one means of doing this, but resources are needed to support their practical implementation in India. Our paper aims to promote the practice of YABs by detailing our experiences, challenges and solutions around hosting a YAB as part of a large mental health project based in Bengaluru, India.

**Main body:**

We hosted a YAB (*n* = 17 members) over three years in Project SAMA (Safeguarding Adolescent Mental Health in India). SAMA was a co-production and feasibility study of a whole-school program to address youth anxiety and depression. We viewed youth perspectives and lived experience as valuable forms of knowledge, and our ambition was for the YAB to enhance the relevance, cultural sensitivity, and effectiveness of our research. A systematic approach was adopted for the recruitment, engagement, and capacity building of YAB members. The team strived to support meaningful engagement and participatory decision-making. The YAB contributed significantly to refining our intervention, improving research tools, and contextualising findings. We identified key challenges and enablers in implementing the YAB in India.

**Conclusion:**

Despite the stigma attached to mental health in India, our study shows that YABs are a feasible platform to support the involvement of young people in mental health research there. Based on our learning, we set out six recommendations to support YABs in Indian mental health research. These recommendations encourage research teams to: identify diversity and representation goals for YAB constitution; delineate the opportunities and boundaries of youth influence on project delivery; allocate sufficient resources to YAB recruitment, training, activities and communication; consider how young people’s learning and accessibility needs and preferences can be ethically surfaced; and plan the monitoring and recording of the YAB’s influence as well as the delivery and evaluation of reciprocal benefits to young people directly.

**Supplementary Information:**

The online version contains supplementary material available at 10.1186/s40900-025-00781-5.

## Abstract (plain language summary)

In many low-and-middle-income countries, including India, the involvement of young people in research has not been taken seriously. There remains a significant gap in engaging youth in meaningful ways in research about them and for them. Guidance is needed to help involve young people more. This paper documents the process of hosting a Youth Advisory Board (YAB) in India as part of a large school mental health project. We report how we used culturally appropriate strategies for recruiting, training and supporting the YAB to support participatory decision-making, regular consultations, and active involvement of youth members. The YAB members played a key role in improving intervention strategies, refining research tools, and contextualizing study findings. Their involvement also strengthened the cultural relevance of the research and improved communication strategies for adolescent mental health initiatives. Through this process, we identified key challenges and enabling factors for implementing YABs in India. This study provides valuable insights into best practices and challenges related to engaging youth in research in India, offering guidance for future initiatives aimed at meaningful youth participation.

**Supplementary Information:**

The online version contains supplementary material available at 10.1186/s40900-025-00781-5.

## Key message

• Adolescent mental health in India is of concern. Investment and innovation in research is needed, shaped by youth involvement and influence.

• Youth PPIE in mental health research is rare in India due to social, institutional and practical barriers. Resources are needed which will progress youth voice in research about them and for them.

• We report our experiences and lessons learned in hosting a Youth Advisory Board as part of a large mental health project in Bengaluru, India, delivered over three years.

• We show that Indian young people are motivated to take part in PPIE in mental health research and are able to add value to research design, delivery and impact. They report personal enjoyment and benefit from participation.

• Youth Advisory Boards are a viable form of PPIE in Indian mental health research and can be very successful with sufficient time, resources, expertise and research team openness.

**Supplementary Information:**

The online version contains supplementary material available at 10.1186/s40900-025-00781-5.

## Introduction

The United Nations Convention on the Rights of the Child (UNCRC) strongly advocates for the rights of children and young people (CYP) to participate, express opinions, and be involved in decisions that affect them, including in research [1]. Such participation rights requires translation into action so that CYP can be catalysts for positive change within their communities [2]. In terms of research, this means conceiving of, inviting and enabling CYP as co-actors rather than passive subjects, where the research is jointly owned and jointly produced by researchers and the ‘researched’ [3]. Patient and Public Involvement and Engagement (PPIE) in research is one way to operationalise participation rights.

Encompassing a wide range of principles and practices, PPIE promotes the rights of the intended beneficiaries of research to be involved in the research work and values their expertise in achieving better research impact [4–9]. PPIE endeavours to be user-oriented, ethical, responsive, needs-led, power-sharing, open to change, open to review, and outcomes-oriented [4, 10–15]. PPIE with adolescents and young people aims to value their insights into their own and others’ experiences and needs which may otherwise be invisible to older researchers, and they are often invited to bring creative solutions to real-world and research problems that affect them directly. To be effective, PPIE in research should strive to be meaningful, defined by the World Health Organisation (WHO) [16] as “an inclusive, intentional, mutually-respectful partnership between adolescents, youth, and adults whereby power is shared, respective contributions are valued, and young people’s ideas, perspectives, skills, and strengths are integrated into the design and delivery of programs, strategies, policies, funding mechanisms, and organizations that affect their lives and their communities, countries, and world.” The WHO is itself just beginning to act on this principle at an organisational level [16–18].

PPIE in research can yield several benefits, including improving recruitment, reducing attrition and power imbalances, mainstreaming marginalised voices, finding practical solutions to complex problems and increasing people’s sense of being valued in their communities [19–25]. It can also improve the appropriateness, quality, generalizability and dissemination of findings [8, 10, 26]. Particular benefits to young people in PPIE have also been reported, including positive relationships with adults, increased self-esteem, a sense of belonging and stigma reduction [27–31]. Yet despite these rights and benefits, PPIE with young people still lags significantly behind PPIE with adults [32, 33] and it is less accepted, funded and validated in some countries than others [3]. Resources are needed to advance youth PPIE in research about them and for them.

Establishing Youth Advisory Boards (YABs) or Young People’s Advisory Groups (YPAGs) or Community Advisory Boards (CABs), who typically represent young people with lived experience or interest in the research topic, is often a first important step in youth PPIE. YABs offer ways for young people to directly and consistently influence research agendas, practices and outcomes from the outset [34, 35], operationalising the ‘early and often’ principle [36, 37] of genuine youth PPIE. They are also a form of PPIE which may be particularly accessible to researchers in terms of format, delivery and routes to impacting projects as they are akin to adult advisory or steering groups in their broad remit. Projects have YABs for varied reasons, in different forms and at different levels. Prior et al. have categorised levels of youth involvement in research from ‘affirming,’ ‘light consultation’ and ‘interactive advice’ to ‘co-production’ and being ‘fully youth-led’ [38].Typical activities in YABs include consulting on research priorities, recruitment approaches, study methods, inclusion practices and dissemination activities.

YABs are sometimes a feature of youth research in high-income countries (HICs), although are yet to become standard practice [38, 39] and face challenges at researcher, organisation and funder-levels [40]. Researchers are often unsure how to logistically, appropriately and creatively engage young people in YABs, organisations often do not offer resources and can present more problems than solutions around ethics and child protection, and funders often do not align expectations of PPIE with the financial, labour and time costs involved in doing it well (although this is changing). Additional challenges to instigating YABs are faced by researchers in low-and-middle-income countries (LMICs), where 90% of the world’s adolescents reside. There are some notable exceptions. For example, e.g. Chow et al. [36] describe Youth Community Advisory Boards in Tanzania, where adolescents living with or affected by HIV collaborated with stakeholders to identify health priorities and inform policies. Hidalgo-Padilla et al. (2025) report on their youth Lived Experience Advisory Panels across Colombia, Peru and Argentina, which worked to influence their research on youth depression and anxiety [41]. Although such studies are beginning to capture the value of youth PPIE in adolescent health research in LMICs, YABs remain rare in LMIC-based youth research.

This is also true in research in India, which is the focus of our work. In India (classified as an LMIC; World Bank 2024) [42], the status and knowledge of adult researchers and professionals are typically deemed sufficient to deliver impactful research, and it is not common for professionals to consult with young people or to be guided by their perspectives. However, this status quo may be impeding research growth as many global research funders expect redressal of the hitherto marginalisation of young people’s lived experience, perspectives, creativity and age-specific knowledge (e.g. youth sub-cultures) in research. To catalyse more youth PPIE in research, Sellars et al.’s (2021) review calls for open reporting of YABs. Although papers are emerging which demonstrate how YABs can be conducted and evaluated in HICs [40], there is limited shared knowledge about practical processes involved in identifying, selecting, training, engaging, remunerating and concluding YABs in LMICs such as India.

Our work has focused on youth involvement in mental health research in India. India has the largest population of adolescents anywhere in the world and their mental health is of serious concern [43]. Nearly 24% of Indian school-going adolescents have a mental health condition [33], mental health stigma is high, the treatment gap for mental health conditions is estimated at 90% [44], and suicide is a leading cause of adolescent death [45, 46] Yet in India (and indeed globally), action for adolescent mental health has been slow and is often shoehorned into adult medico-psychiatric models of practice which do not always reflect or meet the needs of young people [47]. Although there has been increasing global recognition of the importance of adolescent PPIE to energetically and creatively advance mental health support, treatment and research [48, 49], invitations to them to participate in mental health research in India remain rare [40]. There are many possible reasons for this. Clinical service delivery often takes precedence over research, and even in research, traditional hierarchical structures and the social positioning of young people in Indian society undervalue youth participation [1, 42, 50]. Some view Indian young people as politically, economically and socially neglected [51]. Indian governmental and institutional inertia and administrative processes may also be hampering youth PPIE (e.g. low prioritization of youth mental health research; lengthy and risk-averse regulatory processes for involving children and adolescents in research; hurdles in gaining parental consent; slow ethical approvals; red tape tied to state level mental health authorities which prohibit flexibility) [52–54]. In addition, while national programmes such as the Rashtriya Kishor Swasthya Karyakram (RKSK) and the National Youth Policy promote adolescent participation, their translation into structured, institution-level operational frameworks remains limited. There is a lack of trained facilitators and sustained funding for youth PPIE. Finally, logistical challenges with school schedules, travel distances and transport difficulties and digital access gaps makes delivering youth PPIE in India very difficult [55].

The scale of the mental health needs of Indian adolescents demands radical action, which we argue should be heavily youth-informed if not youth-led [33]. Resources to promote purposeful youth engagement in Indian research are lacking. The aim of our paper is to support the growth of PPIE (and specifically YABs) in youth mental health research in India, in ways that respect cultural values in professional identities, hierarchies and social strata whilst actioning the rights of adolescents to influence research about them. This paper describes the practical processes employed in recruiting, training and engaging an Indian YAB for youth mental health research. We detail our processes and discuss challenges, solutions and recommendations for hosting YABs, with particular relevance to youth mental health research in India. Our YAB was based in Bengaluru, India, as part of Project SAMA (Safeguarding Adolescent Mental Health in India), which was a co-design and feasibility test of a whole-school intervention to address anxiety and depression among school-going adolescent children in India [56]. Our intentions for our YAB were that it would bring the views, needs and creativity of Indian young people to promote the quality and impact of our project to benefit young people’s mental health.

## Methods

### Project context

Project SAMA was a three-and-a-half-year international collaboration led by the National Institute of Mental Health and Neuroscience (NIMHANS) in Bengaluru, India and the University of Leeds, UK, along with other Indian and UK partners [56]. NIMHANS is a highly reputable, government-funded centre of excellence in India that delivers research, education, services, and clinical and community mental health treatment. With the ultimate aim of reducing the prevalence of youth anxiety and depression in India, Project SAMA aimed to co-create and feasibility test a whole school mental health program, reaching adolescents, teachers and parents, and addressing mental health literacy, school climate, bullying, help-seeking and reducing the use of teacher corporal punishment. Adolescents, parents, head-teachers, teachers and mental health professionals from local communities contributed to the co-production of the program and associated child safety and program evaluation protocols. In brief, the study demonstrated the appetite for, and feasibility of, delivering a whole-school approach supported by a lay counsellor embedded in the school. Adolescents valued practical psychoeducation to deal with their daily problems and were enthused with new agency to drive school wellbeing from a youth perspective (school climate). Teachers valued the opportunity and space to reflect on their practices and, like parents, felt that understanding adolescent mental health through a developmental and mental health literacy lens diminished stigma and punitive practices. Outcome papers from this study are forthcoming. Ethical approval for Project SAMA, which covered the YAB, was secured from NIMHANS (NIMHANS/26th IEC (Behv Sc Div/2020/2021)) and the University of Leeds (PSYC-221).

### Vision for the YAB

We delivered the project, and our YAB, in line with decolonising research practices which strive to ensure the research is led by and serves the target communities, respecting local knowledge, canons, values, priorities and norms [57]. With regards to our YAB, this meant that NIMHANS would lead its instigation and running, with support as needed from UK partners. Plans for a YAB were built into our funding application with specifications for our timelines (preparation phase and delivery phase to project end), personnel (two Indian YAB facilitators from the project team) and finance (e.g. travel, remuneration, materials) required to instigate and host it. We wanted to establish a YAB from project start to end, functioning at the level of ‘interactive advice’ [40]. Although in some studies YAB members are also the participants in the formal co-production work, this was not the case in our study. We recruited a separate group of adolescents to take part in intervention co-production (full-day workshops) as a way to involve more young people and to not overburden the YAB members. We anticipated that the YAB would bring youth perspectives, needs and ideas to: (i) study design, delivery and dissemination; (ii) solve project challenges; (iii) advocate for action on adolescent well-being; and (iv) hold the project to account (i.e. that we respected, and where possible serve, the needs of Indian adolescents). In return, we anticipated that the project would provide YAB members with an opportunity to: (i) develop skills and knowledge around research and mental health; (ii) meet new and different people outside of one’s usual sphere; (iii) try new things, such as social media creation and speaking at events; and (iv) feel affirmed, valued and useful in bringing youth perspectives to research that responds to real-world issues facing young people.

### Capacity building

As YABs were new to the Indian partner lead (NIMHANS), we allocated time and resources to capacity building which had been costed into the project funding. We held consultation meetings with three professionals highly experienced in running YABs (one from the UK and two from the Centre for Mental Health Law and Policy, India). These consultations surfaced new insights for the project team on best practice and challenges in YABs, especially around key critical processes, such as: recruitment, hosting youth-friendly meetings, incorporating youth suggestions into project delivery, and establishing a stable feedback and communication loop with YAB members. The importance of being able to manage these logistical issues have been also documented in other recent youth PPIE literature [58]. A capacity building phase gave the project team space to reflect and plan what might work well locally in Bengaluru and in NIMHANS. The consultations stressed that YAB members do not need any specialist research or mental health knowledge, as their expertise is in being a young person, potentially with lived experience of a mental health condition.

### Planning the YAB

A lead (MA) and deputy lead (KAL) with experience in working with young people were selected from the SAMA team to coordinate the YAB. They were responsible for recruiting, involving, supporting and communicating with YAB members throughout the project and establishing respectful and affirming working relationships.

Capacity building consultations highlighted the importance of acquiring a good level of diversity within the YAB members and we wanted to have a greater representation of female adolescents given gender inequalities in India and the unique pressures on girls’ mental health and wellbeing. Additionally, a planned effort was made to recruit adolescents from under-represented groups, including young people with disabilities and with diverse sexual and/or racial identities.

The Global Consensus Statement on ‘Meaningful Adolescent and Youth Participation’ and the World Health Organization ‘Toolkit for Adolescents Advocating Change’ informed the formation of the YAB for the SAMA project [16]. These frameworks guided us in designing YAB activities to improve accessibility, inclusion, participation, sense of ownership and empowerment. These frameworks emphasize the importance of establishing a supportive and respectful environment that values open communication, mutual respect, and collaborative decision-making.

After a series of internal discussions, the research team outlined the following expectations for the YAB: (i) to meet once every three months to discuss project progress and critical issues; (ii) to create a WhatsApp group to establish regular communication between YAB members and the research team; (iii) to plan and conduct virtual and in-person meetings following the guidelines of COVID-19; (iv) to support YAB members in devising their own code of conduct and shaping the way they wanted to function to ensure that the YAB remained youth-led; (v) to receive feedback after each meeting on how their views had been incorporated into the project; (vi) to receive annual summary reports from YAB members about their involvement, progress and contribution; (vii) to involve them in social media and filming activities of the project; and (viii) to be involved in dissemination and impact events of the project.

To support the chances of YP securing personally meaningful benefits from YAB participation, the lead and deputy lead received training to deliver the Youth Star tool [59]. This is a fee-based, coaching and outcomes assessment tool, where a youth leader/facilitator helps a young person to identify goals for themselves and measure their ‘journey’ to goal achievement over time. Delivered in a one-to-one format, the tool invites adolescents to consider if they feel ‘stuck’, ‘considering’, ‘having a go’, ‘working on it’ or ‘enjoying and achieving’ across the six life domains of: interests and activities; hopes and dreams; health and wellbeing; education and work; communication; choice and behaviour. We felt this would be a useful tool to help us get to know the young person, to help them derive a broader personal benefit from joining our YAB, and to understand if YAB membership helped them achieve some of their goals. We intended for the YAB leads to administer the tool one-to-one with YAB members at the start, mid-point and end of the YAB.

### Eligibility criteria and recruitment of YAB members

It was decided that YAB members should mirror the main study’s target population, i.e. school-going adolescents, aged 14-18y, living in Kolar or Bengaluru districts in the state of Karnataka. Originally, we planned to form a single YAB with 8–10 members from both districts. However, this was limiting diversity so we opted to created two YAB subgroups, one from each district. We anticipated it would be necessary to cast the recruitment net wide to reach young people who met our inclusion criteria:

- Adolescents aged 14–17 years.
- Lives near Kolar and Bengaluru urban areas.
- Has personal experience of mental health difficulties (with or without accessing mental health service use), or as a carer of someone with mental health difficulties or a genuine interest in matters relating to youth mental health.
- Has ability to listen to others and to express views in a constructive and considerate manner.
- Has ability to work in a team and show respect to people from diverse backgrounds, perspectives, and experiences.
- Has skills related to communication, organisation and time management.
- Has ability to be punctual and reliable.
- Is committed to maintaining contact with the Youth Participation Lead.

An exclusion criterion was current experiencing of serious or complex mental health needs. To identify potential YAB members, key informants across Kolar and Bengaluru were consulted to advise on how to reach young people widely and fairly. Key informants included teachers, NGOs working with schools and communities, the Yuva Spandana program (YSP) and the Life Skills program (LSP). The YSP and LSP programs are youth health promotion initiatives implemented by NIMHANS and the Department of Youth Empowerment and Sports, Government of Karnataka, to promote youth health.

Consultations led to decisions to recruit via: (i) fifteen teachers from various schools in Kolar and Bengaluru, leveraging their close interaction with adolescents to identify potential YAB members; (ii) NGOs working with schools, including SAMVADA, Rainbow Homes, Campus2Community, Sangama, and the Association for Persons with Disability to purposefully recruit under-represented groups, such as youth with disabilities and those identifying as LGBTQ+; (iii) utilization of data from the YSP’s computerized management and information system. This system provided data on 400 adolescents (200 from each region) who had received guidance. As part of YSP, young people had consented to share their contact details to be sent opportunities for adolescents. Permission to access these contact details was obtained from the YSP with clearance from the Institute Ethics Board [60]; and (iv) involvement of 20 Life Skills Trained Officers from Life Skills training and facilitation project, Department of Epidemiology, NIMHANS, who were instrumental in identifying and referring adolescents suitable for YAB participation [61]. In addition, we encouraged nominations from community leaders and peer networks to reach adolescents who might not engage through formal institutional channels. The YAB project leads communicated the YAB eligibility criteria key informants (WhatsApp calls and document sharing). They were asked to approach young people who they felt met the criteria, to briefly explain the YAB opportunity, and to ask consent to pass their contact details to the SAMA project team who could relay further details about the YAB.

A total of 157 adolescents consented to be contacted by phone by the project team. The YSP group were contacted initially via parents (∼ 5 min phone call) who then consented for us to talk their young person there and then; we spent about 5–10 min explaining the YAB opportunity to them and if they were interested, followed up by sending study information material via WhatsApp. This was in vernacular language detailing the eligibility requirements for a YAB member and the application process. Recruitment materials were designed in simple, jargon-free language and shared in multiple formats (written, verbal, and video) to accommodate different literacy levels and communication preferences. Prospective candidates were given 10 days to complete two tasks as a part of the application process. The first task required them to create a 2–4 min video sharing their perspective on adolescent mental health as they perceived it. The second task asked them to write an essay (500 words) on adolescent mental health. These tasks were designed to assess their suitability for the YAB by understanding their interest, views, presentation skills and knowledge of mental health. Although these appear demanding tasks, they were suggested by Indian professionals from the Centre for Mental Health Law and Policy (https://cmhlp.org/) who have experience in establishing YABs for mental health research. These tasks were considered culturally and youth appropriate, leveraging adolescents’ familiarity with digital media and storytelling, allowing them to express their perspectives creatively and authentically. We considered that an essay was a task familiar to them from school and would help us to understand their views on and interest in mental health. The modalities allowed for different communication strengths.

The application process was conducted via WhatsApp. All 157 adolescents initially indicated they would apply with the video clip and essay. A total of 90 applications were received. To reduce selection bias, two independent blinded reviewers assessed applications and evaluated both written and video submissions against pre-defined criteria, namely presentation and communication skills, experience related to, and interest in, mental health, clarity in writing and content relevance. Such rigour in the process was deemed necessary for fairness and transparency. From these, 31 applicants were shortlisted and interviewed online via Google Meet by the YAB Leads. At the point of invitation, we reminded applicants of the exclusion criterion (currently living with a serious mental health condition), as if so, YAB involvement may not be suitable for them but that support could be sought for them if they wanted this. No adolescent declared a mental health condition. Interviews were recorded with the adolescent’s consent at the start of the interview, which lasted approximately 10 min, and explored personal introductions, lived experiences, interest in mental health and the project, likes and hobbies, and reasons for wanting to be in the YAB.

Following this, the project team generated a final list to extend diversity as far as possible. We offered places to 17 members aged between 14 and 18 years, as shown in Table 1, and all accepted. Applicants who were successful were those who conveyed strong interest in collaboration, in mental health, and in representing young people. Applicants who were unsuccessful were contacted with an affirming message indicating they were not successful due to the need to represent people from diverse communities.

**Table 1:** Demographics of our YAB members (*n* = 17)

Domain	Characteristic	*N*
Age on joining (mean, SD)	Mean YAB age	15.29 (1.17)
	Females (*n* = 12)	15.08 (1.11)
	Males (*n* = 5)	15.80 (1.16)
Domicile	Rural	9
	Urban	8
Representation	Past or present mental health condition	4
	Orphaned	2
	Physical disability	1
Kolar Group	Males Females	7 2
Bengaluru Group	Males Females	5 3

### Orientation and training of YAB members

An initial orientation meeting was held for YAB members to meet each other and begin to understand the project; in this orientation, we outlined the broad roles and responsibilities of the YAB members and began discussions about what they might like from participation. This is essential where young people may have assumptions about what they may gain from YAB membership, for example, mental health support (a need noted in other youth health related PPIE work) [58, 62]. We explained how our wider SAMA safeguarding protocol would be applied to the YAB, emphasising the support available for disclosures of child protection issues, how to disclose and the possible steps that would follow.

Given the lack of existing resources, we designed basic training for our YAB members on effective communication, teamwork, research and how YABs can influence it (see Supplementary File Sect. 3 for abroad outline). Support for specific tasks/activities were provided throughout the YAB meetings. YAB members initially expressed a desire to learn about the research process. Hence, the research team shared their expertise in conducting research on adolescent mental health.

### YAB meetings

Monthly meetings (typically 1–2hr) with YAB members were planned to support continual and active youth participation in all aspects of the project. Considerable effort was expended to schedule meetings at time that all YAB members could attend, and these were often in their school holidays and free time. In total, we held six online meetings and five in-person meetings from October 2021 to December 2023. In-person meetings in Kolar were in a room at a private school and in Bengaluru were in a NIMHANS meeting hall. Both had projectors and refreshments and lunch were provided at both sites. Adolescents have busy lives and we sent multiple reminders about upcoming meeting to promote attendance.

A structured format was followed for every meeting, which was important for young people to feel clear about what would be happening each time: *(i) Welcome activity*: a light-hearted, quick check-in activity to give everyone a chance to ‘speak into’ the space (e.g. favourite ice-cream flavour); (ii) *Agenda Review*: the topics to be discussed and the goals for the session; (iii) *Activities and Discussion*: focused on a particular need in the project; (iv) *Reflection period*: A designated reflection period allowed members to evaluate the success of a meeting, identify areas for improvement, and suggest ideas for future meetings. This period was crucial for continuous improvement and maintaining engagement. YAB recommendations included adjustments to meeting timings, modifications in the mode of conducting meetings (preferred in-person meetings), and the optimal duration of each session. The final aspect of the YAB meetings was (v) *Goal Setting*: establishing goals for the subsequent session, providing clear direction and purpose for the group’s ongoing work. Supplementary File 1 (Sect. 4) gives example meeting activities.

The lead and deputy lead were in constant WhatsApp communication with the YAB members, sending friendly messages, reminders and responses. Feedback was sought via WhatsApp after each meeting via open questions (e.g. any thoughts since the meeting? Any feedback for us? ) and or structured questions related to general participation, understanding of the event, meeting dynamics, hearing your voices and impact of the meeting. These were logged by the leads and any follow-up was reported at the next YAB meeting.

The leads also explained how they could support YAB members outside of the YAB. The offer included support with transferable skill development (e.g. presentation skills), educational administrative processes (e.g. form filling), sending information (e.g. around mental health or youth activities) and guidance in seeking mental health support for themselves or others. The leads explained to YAB members that they were open to responding to other requests for support where possible. Two YAB members requested presentation skill development and several asked for guidance about mental health support for someone they cared about. The team responded to these individually.

### Compensation

After internal discussions and meetings with YAB consultants, it was agreed to pay YAB members Rs. 500 ($5.78, £4.74) per meeting, reflecting local pay rates for youth work. Payments were made directly from the NIMHANS account as a demand draft/account transfer in the name of the YAB member. Travel expenses for YAB members and any chaperones to in-person meetings were reimbursed on presentation of receipts and completing an expense claim form.

### Challenges in running the YAB

The NIMHANS team hosting the YAB reminded the project team that youth voices are often overlooked in Indian culture and that this presented two main challenges for our YAB work – first for the adolescents to adapt to, and trust, that the project did genuinely seek their views, and second, for the facilitators to adopt a counter-cultural mindset and be fully receptive to adolescents’ perspectives. However, adaptation on both parts occurred quickly (by meeting 2), as relationships and new ‘rules of engagement’ emerged. Notably, during the orientation and training, we noted rural YAB members were more hesitant to speak than their urban counterparts, so we switched to small group discussions first and rotated speaking opportunities. Cultural inclusion was fostered by incorporating local languages where possible, respecting regional customs, and ensuring examples reflected diverse rural and urban realities. These measures gradually built confidence among quieter participants.

The onset of COVID-19 necessitated holding initial meetings online. This presented difficulties in quickly establishing rapport with YAB members. Some members later reported feeling unable to connect with each other early on, and also felt they were holding back until they had got a sense of how trustworthy and safe the group was. It was also hard in an online meeting to support vibrant exchanges of perspectives and ideas, and leaders reflected that initially they themselves were not confident in how to facilitate connection and high engagement in an online group. This was redressed by asking the YAB for solutions, and with support from the research team, in restructuring the meetings and identifying tasks that would work well online (e.g. ranking tasks or watching brief videos to spur debate). Limited access to mobile devices and stable internet connectivity among some members further hindered consistent participation during the early stages of the YAB. The in-person meetings were experienced as more energetic, productive and enjoyable.

A further challenge was that early tasks appeared too taxing or unrewarding especially when it involved lengthy periods of listening to each other (e.g. generating an age-appropriate glossary of key mental health terms). Asking adolescents to read documents in advance (e.g. our draft safeguarding protocol) was also ineffective as there were diverse levels of engagement and understanding. We quickly adapted to ensure every YAB task has a very easy entry-point, did not overlabour discussions, and involved fun and creativity as often as possible. This required careful navigation of the needs of the project, the pursuit of influential youth voice, and the delivery of a positive experience for all YAB members.

### How our YAB impacted project SAMA

The dynamic interaction between the YAB members and the YAB facilitators, which emerged after the first few meetings, cultivated a mutually enriching exchange of ideas and expertise, which enthused and helped the research team feel confident that broader project decisions would meet the needs of many young people. Table 2 gives examples of the tasks and impacts of the YAB on Project SAMA and demonstrates how the members engaged with multiple strands of the work, from aspects of research delivery to co-production to dissemination.

**Table 2:** How the YAB members assisted and influenced project SAMA

Project Area	YAB Task	Impact on Project
Cross-project	Sharing examples of everyday life as an Indian school-going adolescent.	Kept us attuned to the realities of their lives, which challenged us to consider where mental health interventions might credibly be helpful; created a store of examples to convey back to study schools.
Refining intervention vocabularies	Discussed a list of key words (e.g. mental health conditions, self-compassion, stigma) and their suitability for use in the intervention.	For example, changed our use of key terms (from ‘mental health’ to ‘wellbeing’, from ‘stress’ to ‘tension’). Prompted us to define some terms (e.g. stigma) using real-life examples and to avoid use of those which young people felt were not relevant to them. For example, although self-compassion has been reported as highly relevant to some young Indian people in the context of mental health [63], and we hoped to draw on the concept as part of our youth-focused intervention, our YAB indicated that the term was not familiar to them, nor one they could practically use.
Safeguarding Protocol	Reviewed the planned procedure for the research team to act on child safety concerns, including bullying.	Confirmed acceptability of the overall plan, but with refinements to the emphasis on what could be private and what must be shared with other professionals.
Intervention content (youth)	Review some of the major areas of the intervention youth-focused component and identify where planned content or activities might not work or could be optimised to be more youth-appropriate, fun and helpful.	Endorsed most content and activities, which gave the project team confidence in the likely intervention acceptability. Charged the project team with being consistent in using relevant examples from Indian adolescent life and with emphasising the practical applications of intended learning.
Intervention content (parents)	Creatively suggested ways to bridge adolescent-parent communication barriers	Incorporated principles into study information letters and the parent intervention component.
Training of delivery agents	Evaluated some of the content of the planned training for delivery agents.	Changed some content to connect more with contemporary Indian adolescent life; increased our focus on the importance of delivery agents being self-aware of their own values and respecting differences in young people.
Intervention manuals	Reviewed some of the draft manuals to identify where the guidance may not be sufficiently youth-centred.	Changed some of the terms and messages in the intervention manuals to ensure that the principle of youth voice was a consistent priority; removed content which unintentionally appeared to value some youth attributes over others.
Standardised measures	Identify likely challenges in completing measures of youth outcomes	Challenged the research team to reflect on assumptions that adolescents understand research data as distinct from school assessment. Changed our administration plan to multiple sessions, reading items aloud and more clearly explaining the purpose of the questions and use of data.
Dissemination and advocacy	Assisted the design of some dissemination / impact events and spoke at those	Challenged us to more invigorating and ambitious calls for action and more confident dissemination of the project’s impact.

Table 2 highlights three important aspects of the YAB’s influence on the project. First, the YAB advocated strongly for our program to be consistently practical and grounded in the everyday realities of their lives. Second, the YAB revealed to us how aspects of the project (e.g. data collection, lay counsellors) might be perceived differently by adolescents versus adults and the need to see research processes from a young person’s perspective. Third, the adolescents’ passion for change and action, and their endorsement of the program, highly motivated and energised the research team.

In addition, the fact that our project was delivered in close collaboration with adolescents (both in the YAB and intervention co-design) appeared to be a highly attractive feature to both our delivery agents, study schools and young people who were study participants. It appeared to confer greater confidence to them that the program would be acceptable to young people, would speak to their needs and would have a level of novelty and youth creativity.

### Concluding the YAB

We discussed with YAB members about their preferences for our final session, and they asked for time to discuss skills to manage their daily difficulties. Two experienced mental health researchers facilitated life skills sessions with the YAB, focusing on managing emotions, decision making, interpersonal relationships and stress management. To formally and publically recognise and celebrate the work of the YAB in Project SAMA, all YAB members were invited to NIMHANS; we felt it was important to invite them to the Institute rather than hold a school-based event to foster their sense of place and belonging in professional and civic life. The final celebration event included summaries of the work and impact of the YAB and expressions of gratitude; all received a small memento and were thanked formally for their contribution.

### Evaluating the benefit of being a YAB member

At project end, YAB members were asked to share, via an anonymous survey distributed via a link in WhatsApp, their experiences of being part of the YAB. The survey was an open text form where they could detail as much or as little as they wished about their experiences, any benefits of being on the YAB and any other feedback for the team. Of the 17 YAB members, 8 females and 2 males responded, and outcomes are summarised in Table 3. They expressed positive outcomes in four domains; greater knowledge and understanding of youth mental health; communication, problem-solving and idea generation; personal development (emotionally and cognitively); and diverse benefits linked to novel opportunities. Whilst we had anticipated that YAB involvement might foster improved confidence in communication and self-expression (termed ‘voicing out’ in India), we had not anticipated improved self-awareness and wellbeing nor the extent to which their own knowledge and understanding of mental health would grow. Most non-respondents were male and from rural backgrounds. Non-response may be attributed to factors such as limited digital access, low familiarity with online survey formats, and hesitation in sharing views in a written format.

**Table 3:** Perceived benefits of YAB members (*n* = 10 respondents)

Reported benefits	*N*	Indicative quotes
Learning about youth mental health and wellbeing	10	• I developed a deeper understanding of mental health and its importance. We got to know about depression, anxiety, bullying and we can recognize and address such issue in the future. • After being a part of SAMA YAB, we all know the importance of our well-being. […] At starting, we had a little bit of knowledge about mental health. After SAMA, we come across different types of issues which people are facing. • SAMA provided us awareness of mental health, which is very important because it helps so many students overcome depression, anxiety, and other challenges. • It made us feel we were not alone and the problems were common among adolescents. • Our understanding of mental health and the importance of mental well-being are widened
Greater confidence in communication, problem-solving and expressing views and ideas	8	• Workshops and activities enhanced our thinking and communication skills. Our problem-solving skills were improved. Interactive sessions helped us to be confident in sharing and developing ideas. • The YAB mainly provided me the opportunity to share my diverse opinions on various situations and questions. • It gave us a lot of confidence in the sessions we used to share our thoughts without any hesitation. • SAMA created a safe platform for us to freely express our opinions and ideas. We felt valued, heard empowered. • I learned conversation skills and how to apply new learnings in life. • The innovative activities […] enhanced my skills, confidence, and awareness
Personal development (cognitive, emotional and life skills)	6	• The YAB activities helped us to understand and manage our moods better. We gained more self-control, developed peace and felt more relaxed and balanced. • I learned to manage my stress and improve my emotional well-being through SAMA sessions. • The activities conducted by team SAMA helped us to develop practical skills in handling challenging life events. We improved our confidence to apply learning in our real life. • My concentration power had increased due to the activities which were given. • The workshops provided by SAMA enabled me to develop my skills in thinking and communication.
Experiencing important opportunities and further motivation to help	6	• We got an opportunity to interact with teachers, policymakers, social activists and international participants. It helped us to widen our connections with professionals. • SAMA provided me the opportunity to interact with foreigners engaged with the program. • Being part of YAB gave me opportunities to spread awareness, and showcase my talents. • SAMA enhanced my interest in further participation in these activities. • We got incentives for our participation and contribution. This motivated us to stay committed and feel appreciated for our efforts. • Being involved [helped me to] learn how to interact outside the world.

## Discussion

To galvanise effort towards the involvement of young people in research about them and for them, there are repeated calls for publications on practical ways to do this, including reports on establishing and administering YABs [40]. PPIE in youth and mental health research in India is rare. Our paper aims to promote the practice of YABs by detailing our experiences, challenges and solutions around instigating and running a YAB as part of a large mental health project based in Bengaluru, India. Here, we focus on six key findings from our work, reflecting on what worked well, challenges and solutions, and recommendations for researchers considering hosting YABs, with particular relevance to India.

First, our findings show that it was feasible to engage Indian adolescents in a topic (mental health) that is highly stigmatised in India [64], and to sustain their engagement over a long period of time (three years). The high number of applications to our YAB suggests that many Indian adolescents (at least in our target area) found this opportunity attractive and sufficiently so to undergo an application and interview process. We did not encounter any difficulties in the YAB’s willingness to discuss mental health.

Recommendation 1: Do not assume that cultural stigma around certain topics will mean YABs in India are not feasible. Assume that there will be youth interest in research and that some adolescents will be willing to share lived experience or broader views on sensitive subjects.

Second, designing and delivering the recruitment and selection process of our YAB was extremely time- and resource-heavy. Studies involving YABs do not always report their recruitment and selection processes. It may appear that our processes were burdensome and potentially stressful for young people. However, we opted for this approach given the experience of a third sector organisation in India (Centre for Mental Health and Policy) who encouraged us to anticipate a high volume of applications and the need for a transparent, auditable and supportive selection process to ensure young people appointed to the YAB have sufficient motivation to remain engaged over a long project. This is important as continuity matters when a group will have established connections and new member assimilation can be tricky, and topping up recruitment of YABs takes resources. Ours is only one example of how to recruit for YABs and many other processes may work in India and elsewhere. We strived to recruit a diverse sample, and to at least have representation around disability and with more females than males, because evidence shows they have distinct mental health experiences [65–67]. We met our gender target but had low diversity in sexual and other identities. The nature and extent of diversity required in YABs will differ for different projects. Research teams should remember that whilst the composition of a YAB matters, no YAB can represent all young people. Research teams should balance their YABs’ perspectives with the wider knowledge they may hold about other sub-groups (as also argued by [68].

Recommendation 2a: Resources should be allocated to ensure equitable and transparent appointment of YAB members who understand the required commitment.

Recommendation 2b: Projects should consider in advance what will be sufficient diversity in their YAB given their research aims.

Third, the principle of PPIE means that efforts must be made to ensure young people can contribute and influence research. Providing training to YAB members is one way of doing this, where training is defined as the development of understanding and/ or specific skills for application to project delivery. Although some researchers caution against extensive training of PPIE contributors, as it risks professionalising them in ways that means they no longer represent the intended grassroots beneficiaries [3], a lack of training can be a barrier to meaningful contribution, as experienced in some studies [41]. Shared resources or standard practices for training youth for PPIE, including YAB membership, are rare meaning researchers expend significant effort crafting their own training materials or inviting guest speakers. We had to generate our own training for our YAB members; we describe this as a ‘best effort’ and we remain unsure about how to judge the sufficiency of such provision which was by a small team, over a long project and for a diverse group of young people with different needs and expectations in joining the YAB. Published reports of how others have trained YAB members (in the context of child health research and practice) range from none to extensive training on topics such as qualitative and quantitative research methodologies and knowledge translation and patient safety [5]. Sharing of high-quality training materials for YABs which could be adapted to culture, age and context, would likely have a significant impact on a research team’s sense of efficacy to set-up YABs as well as securing more meaningful engagement from young people. Training could helpfully cover: the purpose and potential of research, foundational research methods, ethical requirements, use of existing evidence, pathways to impact, and the role of dissemination and advocacy. Given that many YABs will meet online and investing in researcher and YAB training on virtual communication and digital tools, which include how to build a group connectedness online, could optimise PPIE.

Recommendation 3: Research teams should allocate sufficient time and resources to preparing YAB training materials and, if effective, should share these as widely as possible.

Fourth, time and funding are core organisational barriers to achieving meaningful youth participation in mental health research [69]. Successful youth PPIE requires age-appropriate and engaging ways for young people to contribute their views, creativity and solutions [70, 71]. Designing these activities needs time, skill and enthusiasm. As in other studies [72], and in line with recommendations for sufficient resource allocation [73], our project benefitted in that we had planned for a period of learning (capacity building) and had allocated two project staff to manage the YAB, including creating modes of effective and sustained engagement. Our investment here, and continual communication with our YAB members, may have insulated our YAB from the levels of attrition reported by others (e.g. Hidalgo-Pardilla et al., 2025). The initiation of our YAB was planned during the COVID-19 pandemic and we faced initial challenges in that virtual meetings did not facilitate dynamic idea exchange. Our YAB members reported initial difficulty in opening up and bonding, and had concerns related to privacy and technical glitches, difficulties also reported by others [41]. A collaborative solution-focused approach was used to overcome these and YAB members gradually acclimated to the digital platform. However, the in-person events always appeared more energetic and rewarding for YAB members.

Although the team had considerable experience in youth PPIE in the UK [74, 75], the translation of successful PPIE activities from there to India was not straightforward. Differences in cultural expectations around the extent to which adolescents can voice opinions, their familiarity with creative activities and their sensitivity to hierarchy meant the typical ‘rules of engagement’ between researchers and young people in PPIE did not always work. We had to adapt ways of working to slowly build a working relationship with the YAB where we could demonstrate their ‘voice’ and creativity was welcome, secure and needed. Additionally, as is common in PPIE activities with young people [76], early tasks for the YAB were sometimes too difficult or non-engaging, especially when it involved a considerable amount of reading or lengthy discussions. Unsurprisingly, young people responded best to creative, participatory methods and least enjoyed tasks based on reading documents or having to absorb a lot of information (manifested as low responsivity or requests for alternative tasks). We therefore adapted tasks to require only short amount of reading, distilling key points for them and giving space for creativity and not just discussion. The type of activities are significant in YABs [75]; certain activities may unintentionally reproduce power imbalances and exclude youth (e.g. via the use of jargon or focusing on project issues where youth can have little influence). Using participatory tools like visual mapping or storytelling might have engaged our YAB more effectively. Reviews are underway to begin to capture the range of activities being used in youth PPIE [77].

We also should have enquired about learning needs among our YAB members (e.g. related to autism, dyslexia or ADHD) and given them the opportunity to advise us on how we could make accommodations to support their meaningful engagement. However, making such enquiries in India is complex [78, 79], as the understanding and assessment of this leaning needs is relatively poor, and there remains stigma around disclosure [64, 80]. Finally, our named YAB leads were crucial in facilitating YAB meetings to ensure discussions remained relevant and useful for the research.

Recommendation 4a: Projects should allocate resources (staff, time and money) to adequately prepare for YAB meetings and activities to ensure young people can contribute to their full potential.

Recommendation 4b: All YAB members should be invited to privately convey to the research team any learning or support needs to ensure project tasks are accessible to them.

Fifth, working with our YAB directly influenced many aspects of our thinking and understanding, as well as project delivery. Although not easy to quantify or evidence, we as researchers felt more attuned to, and therefore accountable to, young people who were our ultimate project beneficiaries. Our conversations with them kept our work alive to the realities of their lives, continually invigorated our purpose and imparted significant confidence to the importance of our work. Our paper also reports the concrete impacts on project delivery, informed by the adolescents’ insights into their own well-being and mental health experiences, as well as expert knowledge on life as an Indian adolescent. Other studies similarly report that young people can reflect on and bring lived experience knowledge to helpfully advise research [81] and are a step towards redressing the exclusion of young people from research on matters most to them, including mental health [41, 82]. As with many other studies, our work shows that YABs can be platforms for young people to impact research if research teams are genuine about their willingness to be shaped by young people and are prepared to go against ‘received wisdom’, academic knowledge or social hierarchies around whose opinion matters most. A key principle of PPIE is that research teams need to be genuine about flattening hierarchies and about learning from and with young people, not dismissing their views as naive and to not assume that academic knowledge is always sufficient, complete and accurate. Working with these values and processes can mean reconfiguring what it means to be a researcher vs. the researched or expert vs. novice. Furthermore, as ‘making a difference’ is a key value for young people who come forward for research participation [73, 83] studies running youth YABs should ensure the young people themselves are clear how the giving of their time, energy and perspectives has made an impact.

Recommendation 5a: in advance of YAB instigation, research teams should prepare a draft concordat (to be consulted with young people) which sets out how, and to what extent, the project may be able to respond to youth voice. This is especially important for multidisciplinary teams who may have different experiences and expectations about the influence of PPIE in research.

Recommendation 5b: to systematically record the way the YAB were involved in the main project and how their perspectives shaped the research in order (i) demonstrate the impact to young people themselves and (ii) to publish this to advance the understanding of the impact of youth PPIE.

Finally, when YABs are well managed, they can be very positive experience for young people. Nonetheless, like other studies with YABs in other countries and research areas [38, 41], our YAB members reported a number of personal benefits from learning about mental health, to self-development and skill building, but also to a sense that they were contributing to the real-world challenge of youth mental health. This reflects global findings. In a large survey with over 40,000 15–29-year-olds, young people from five LMICs reported that they felt they could make the greatest impact by being part of projects that raise awareness about mental health, and especially via school-based initiatives [81]. YABS can be positive civil society experiences for Indian young people, an important finding given that opportunities for youth civic engagement are few, despite national ambitions for greater youth participation [84]. PPIE can be an effective way for often disenfranchised youth to engage civically. To deliver such benefits from PPIE to young people, support is crucial. Watson et al.’s (2022) review of the impacts of being involved in mental health research in the UK reported that, underpinning all of the benefits recounted by young people, was the importance of researchers allocating sufficient time and resources to support them, e.g. in forming meaningful relationships, managing expectations and providing trusting and safe environments for them to talk openly.

Evidencing the impact of YAB participation for young people remains a priority. We had ambitious plans to administer the Youth Star (https://www.outcomesstar.org.uk/) in one-to-one meetings, assessing the personal development goals of each YAB member at project start, midway and at project end to review goal achievement. However, we faced insurmountable difficulties largely due to the disruption caused by COVID-19 at project start, meaning we could not meet the YAB members in person to administer the tool, and there was a lack of supportive digital tools and team expertise in remote administration of the tool. Whilst gathering qualitative feedback on experiences in YABs should remain a legitimate way of exploring their impact (e.g. Hidalgo-Padilla et al., 2025), future research could also evidence outcomes by administering pre-post evaluation instruments, guided by the outcomes valued by the young people themselves, to promote standardisation and comparison across YABs and different ways of operationalising them.

Recommendation 6: Plan how the research team will identify how benefits could be secured for YAB members and incorporate delivery of those benefits into the YAB from the outset. Evaluate the experience of being a YAB member to improve future practice.

Following their first experience of hosting a YAB, NIMHANS plans to initiate youth participation in institutional governance by integrating YAB members into policy and project advisory committees. They hope to involve YAB in co-creating future projects, enabling adolescents to shape program design and evaluation, and to strengthen youth leadership and intergenerational collaboration through dedicated capacity-building initiatives.

### Limitations

Our work should be considered in the light of a number of limitations. Representation on YABs, as in all research, remains a challenge, especially in a country as diverse as India. In reality, the particular group of young people in any YAB (as with other advisory groups and research participants themselves) can only be marginally representative of the target population of the research. Nonetheless, our intention to recruit often excluded young people to our YAB was not as successful as we had hoped, and we do not fully understand the reasons why we did not attract representation from more marginalised groups. The best that can be done in such circumstances is to consider the possible impacts of the particular demographics, and positionality, of the YAB members that were recruited – but this will always be speculative. It is likely that, as our YAB were made up of more girls than boys, that some influences on Project SAMA were gendered. However, we did not observe any stark differences between genders in our discussions. It is possible that as we did not recruit young people who identified as gay or transgender that issues pertaining to those identities were not made as visible.

Our paper is a self-report of our experiences in creating and running a YAB in India. The quantity of feedback data from our YAB members was small, and although anonymous, may be subject to self-selection and reporter bias. We believe that feedback from the YAB members was restricted by their own academic workloads, a cultural preference for verbal over written expression, and reluctance to offer critical comments to perceived authority figures. Additionally, translating ideas into structured written feedback was challenging for many due to limited reflective writing experience. Future studies could consider asking YAB members to interview each other, and to utilise other means of evaluation to ensure YAB members have safe ways to be critical of a project’s processes or to report other concerns. We could have used semi-structured interviews, observation notes, and brief debriefing sessions after meetings to capture richer feedback. Finally, the generalizability of learning from our study may be limited by the specific sociocultural and geographic contexts of the participating schools.

## Conclusion

Opportunities for youth participation in mental health research in LMICs such as India, remains scare [40]. Establishing and managing a YAB under the SAMA project at NIMHANS constituted a step forward in youth PPIE for this important Indian institute. Our work demonstrates that YABs are a highly feasible and effective way to promote the voice of young people in youth mental health research and may be one way to promote the civic engagement by young people in India, with multiple benefits to young people and research [84]. We call for greater youth involvement in Indian research to progress radical action for adolescent wellbeing, and endorse calls for PPIE which extends not just to youth involvement, but to youth leadership [82] in this field.

## Supplementary Information

Below is the link to the electronic supplementary material.

Supplementary Material 1

Supplementary Material 2

## Data Availability

No datasets were generated or analysed during the current study.

## Abbreviations

YAB: Youth advisory board
LMIC: Lower Middle-Income Countries
HIC: Higher Income Countries
PPIE: Patient People Involvement and Engagement
WHO: World Health Organization
UNCRC: United Nations Convention on Rights of the Child
CYP: Children and Young People
YPAG: Young People Advisory Groups
CAB: Community Advisory Boards
SAMA: Safeguarding Adolescent Mental Health in India
