# Supplementary material for: Progressing participatory research with young people in India: how a youth advisory board supported a large mental health project in Bengaluru
Source: Res Involv Engagem. 2025 Oct 14;11:116. doi: 10.1186/s40900-025-00781-5 (PMC12522547; doi:10.1186/s40900-025-00781-5)
Supplement: Supplementary file 1 — Supplementary Material 1 [file 40900_2025_781_MOESM1_ESM.pdf]

ADDITIONAL FILE

Section and topic	Item	Reported on page No
1: Aim	Report the aim of PPI in the study	3
2: Methods	Provide a clear description of the methods used for PPI in the study	7-15
3: Study results	Outcomes—Report the results of PPI in the study, including both positive and negative outcomes	16-25
4: Discussion and conclusions	Outcomes—Comment on the extent to which PPI influenced the study overall. Describe positive and negative effects	27
5: Reflections/critical perspective	Comment critically on the study, reflecting on the things that went well and those that did not, so others can learn from this experience	23-29
