# Supplementary material for: Progressing participatory research with young people in India: how a youth advisory board supported a large mental health project in Bengaluru
Source: Res Involv Engagem. 2025 Oct 14;11:116. doi: 10.1186/s40900-025-00781-5 (PMC12522547; doi:10.1186/s40900-025-00781-5)
Supplement: Supplementary file 2 — Supplementary Material 2 [file 40900_2025_781_MOESM2_ESM.pdf]

What is this manual? ....Error! Bookmark not defined.

Shared Learning *Hosting a Youth Advisory Board in* Project SAMA

Project SAMA was jointly led by the National Institute of Mental Health and Neurosciences (Bengaluru, India) and the University of Leeds (UK)

This work was supported by the UK's Medical Research Council, Economic and Social Research Council, National Institute of Health Research and UK Aid (Grant number MR/T040238/1

Project SAMA 2
Youth Voice in Project SAMA 2
1.0 Preparing to host a YAB 3
1.1 Funding 4
1.2 Staff capacity building 4
1.3 Team agreement on key aspects of the YAB 4
1.3.1 Broad Vision 4
1.3.2 Leadership and communication 5
1.3.3 Considerations regarding representation 6
1.3.4 Renumeration 6
2.0 Recruitment and Retention 6
2.1 Eligibility Criteria 6
2.2 How we recruited 7
2.3 How we selected 8
2.3.1 Application process 8
2.3.2 Interview process 8
2.3.3 Final appointments considering diversity 9
3.0 Orientation, training and 'baseline' measures 9
3.1 'Baseline' measures 9
3.2 Orientation and training for YAB members 10
3.3 Setting up meetings 12
4.0 The YAB meetings 12
4.1 Following a format 12
4.2 Recording meetings 13
4.3 In-between meetings 13
4.4 Example meetings 13

2025

Written by Professor Siobhan Hugh-Jones (University of Leeds, s.hugh-jones@leedsa,cuk) and Dr Mutharaju Arelingaiah (NIMHANS)

What is this document for?

This documents our processes, reflections and recommendations about running Youth Advisory Board (YABs) as part of Project SAMA in India. We hope it can support future researchers in hosting YABs to increase the inclusion of adolescents and young people as research advisors or co-researchers in research about them and for them.

Project SAMA

SAMA was a three-year research project that worked with adolescents and other stakeholders to co-produce and feasibility test a whole school program to support the emotional well-being of school-going adolescents in India.

Using best evidence, and co-production, the program aimed to provide Indian adolescents with information and strategies to take care of their emotional well-being and to improve school cultures, including teacher and parent mental health literacy to support the well-being of its young people. See www.sama.org.uk for more details.

Youth Voice in Project SAMA

Adolescents have remarkable potential to be creative agents of change in their schools and communities. Youth participation was central to Project SAMA. The purpose of the SAMA YAB was to ensure effective and successful delivery of the project's main aims and interventions by listening to the views of young people to shape project decisions.

Partnerships between researchers and adolescents in research is increasing in popularity. Adolescents are often experts in their own right by lived experience, which can refer to any adolescent with experience in the topic of the research. Progressively, many researchers are now responding to the call for 'no research about me, without me' meaning decisions that directly affect a participant group should not be made without consulting that population group.

The involvement of adolescents in research, as co-researchers or consultants, can bring mutual benefits to adolescents themselves, to the research itself and to researchers.

Young people may benefit by:

- gain new knowledge and skills.
- broaden their social skills and connections
- increase their independence, confidence and self-esteem.

The research may benefit from:

- being more relevant to the needs and experiences of young people. Youth can provide valuable insights into the challenges they face and the responses and support that are most likely to be effective. By involving youth in the research planning and design process, researchers can ensure that the research questions or hypotheses are relevant and meaningful to youth.
- a sense of ownership and buy-in for the interventions being developed. When youth are involved in the research process, they feel invested in the outcomes and are more likely to engage in the interventions being developed. By involving youth in the intervention development process, researchers can ensure that the interventions are relevant, engaging, and effective for youth.
- promoting the validity of the research. Youth can help ensure that the research methods and instruments are appropriate and sensitive to their experiences.
- promoting a culture of research and evidence-based practice among youth. By involving youth in the research process, they learn about the importance of research and the role it plays in improving the lives of youth. This can help promote a culture of research and evidence-based practice among youth, which can lead to more effective interventions and better outcomes for youth
- supporting the dissemination and impact of the research findings. Youth can help disseminate the research findings to their peers and communities in ways that are engaging and relevant. By involving youth in the research dissemination process, researchers can ensure that the research findings are shared with the people who can benefit from them the most.

Researchers may benefit from:

- an increased understanding about the adolescent perspective and enhanced data collection methods.
- feeling motivated and encouraged that the work is meaningful
- being encouraged to be creative, take risks and question received wisdom

1.0 Preparing to host a YAB

Our approach to the YAB of Project SAMA was informed by the Global Consensus Statement on Meaningful Adolescent and Youth Participation and the World Health Organisation Toolkit for Adolescents Advocating Change.

1.1 Funding

YABs incur costs (e.g. paying young people, travel expenses, subsistence, room hire, materials, training). Sufficient funds should be sought to ensure the YAB is well resourced.

We included costs for a YAB in the funding application for Project SAMA to cover staff training, renumeration to young people, their travel (plus that of a chaperone if needed), materials and refreshments.

1.2 Staff capacity building

YABs take time, creativity and capacity to run well. It can help to appoint staff who have a dedicated responsibility to the YAB, and who will enjoy working closely and in partnership with young people.

From our research team, we appointed a Lead and Deputy Leads for the YAB. They were given the responsibility for planning, recruitment, retention and coordinating/ planning YAB meetings and activities. Both had experience of working with young people and were keen to take up this role

As YABs were new to NIMHANS, we allocated time and resources to capacity building which had been costed into project funding. We secured some consultation and guidance from and Indian and UK partner who had experience of running YABs:

- Niyah Campbell, (University of Birmingham, UK)
- Jasmine Kalha (Centre for Mental Health Law and Policy, India)
- Swetha Ranganathan (Centre for Mental Health Law and Policy, India)

They helped us to gain insight into the processes of:
- Recruiting YAB members
- Retaining YAB members
- Arranging and hosting meetings
- Implementing actions
- YAB feedback loop

1.3 Team agreement on key aspects of the YAB

1.3.1 Broad Vision

Agreement as a research team about the purpose, vision and operation of the YAB – and what the YAB members can expect in return – is important to establish in advance. Especially important are principles by which the research team will respect and respond to the YAB's views, requests and recommendations in the research and in

the YAB itself, and how lines of communication will operate within the YAB and with the research team.

Our intentions for our YAB were that it would bring the view, needs and creativity of Indian young people to promote the quality and impact of our project to benefit young people's mental health. We agreed that we would:

- support our YAB by getting to know them as individuals, helping them identify their strengths and learning about how they would like to contribute to the YAB.
- identify any training and information needs they may have, and work with them to ensure they feel supported.
- run the YAB from project start to project end.

Specifically, we hoped that our YAB would bring youth perspectives, needs and ideas to:

- study design, delivery and dissemination
- solve project challenges
- advocate for action on adolescent well-being
- hold the project to account (i.e. that we respected, and where possible serve, the needs of Indian adolescents)

In return, we anticipated that the project would provide YAB members with an opportunity to:

- develop skills and knowledge around research and mental health
- meet new and different people outside of one's usual sphere
- try new things, such as social media creation and speaking at events
- feel affirmed, valued and useful in bringing youth perspectives to research that responds to real-world issues facing young people.

1.3.2 Leadership and communication

We agreed that

- the YAB staff leads would report on the YAB at every research team meeting, and that the research team could invite youth voice at any point via the YAB leads.
- The YAB would use WhatsApp for their communications
- The YAB leads would regularly update the YAB members on how the research was responding to their input

We set out expectations of the YAB as follows:

- Meet on a quarterly basis
- Be in frequent communication via YAB WhatsApp group.
- Meet virtually (due to COVID-19) then in person when safe to do so.

- Be responsible for shaping how they want to work as a board – devising their own code of conduct and agreeing their approach to governing the research project to ensure it is youth-led.
- Provide feedback and recommendations after each meeting for the next one.
- Contribute to dissemination and impact work

1.3.3 Considerations regarding representation

Internal discussions highlighted the importance of acquiring diverse representation within the YAB. We wanted to recruit a greater representation of female adolescents to reflect the additional societal pressures this gender faces and the influence this has upon the mental health of female adolescents. Since the project is focussing on strengthening adolescent mental health, it was considered critical to capture very practical experience of adolescent lived experience by involving such them in the YAB. Additionally, a planned effort was also made to involve adolescents belonging to special groups such as those with disability or of a third gender.

1.3.4 Renumeration

After many internal discussions and meetings with YAB consultants it was agreed that YAB members would be remunerated at Rs. 500 per meeting (in line with the budget allocated within the grant). The payment would be done directly from the NIMHANS account as Demand Draft in the name of YAB member. In addition, we agreed travel expenses of members and accompanying persons would be reimbursed and that refreshments would be provided.

2.0 Recruitment and Retention

2.1 Eligibility Criteria

It was decided that YAB members should mirror the main study's target population, i.e. school-going adolescents, aged 14-18y, living in Kolar or Bengaluru districts in the state of Karnataka. Originally, we planned to form a single YAB with 8-10 members from both districts. However, this was limiting diversity so we opted to created two YAB subgroups, one from each district. We anticipated it would be necessary to cast the recruitment net wide to reach young people who met our inclusion criteria:

- Adolescents aged 14–17 years.
- Lives in places near Kolar and Bengaluru urban areas.
- Has personal experience of mental health difficulties (with or without accessing mental health service use), or as a carer of someone with mental health difficulties or a genuine interest in matters relating to youth mental health.

- Has ability to listen to others and to express views in a constructive and considerate manner.
- Has ability to work in a team and show respect to people from diverse backgrounds, perspectives, and experiences.
- Have skills related to communication, organisation and time-management.
- Has ability to be punctual and reliable.
- Is committed to maintaining contact with the Youth Participation Lead.

An exclusion criterion was current experiencing of serious or complex mental health needs.

2.2 How we recruited

Key informants across Kolar and Bengaluru were consulted to advise on how to reach young people widely and led to decisions to recruit via:

- fifteen teachers from various schools in Kolar and Bengaluru
- NGOs working with schools, including 'SAMVADA', 'Rainbow Homes', 'Campus2Community', 'Sangama', and the 'Association for Persons with Disability', to purposefully recruit under-represented groups, such as youth with disabilities and those identifying as LGBTQ+
- utilization of data (with consent) from Yuva Spandana Program (Computerized Monitoring and Information System)
- involvement of 20 Life Skills Trained Officers from Life Skills training and facilitation project, Department of Epidemiology, NIMHANS,
- we encouraged nominations from community leaders and peer networks to reach adolescents who might not engage through formal institutional channels.

The YAB project leads communicated the YAB eligibility criteria key informants (WhatsApp calls and document sharing). They were asked to approach young people who they felt met the criteria, to briefly explain the YAB opportunity, and to ask consent to pass their contact details to the SAMA project team who could relay further details about the YAB.

In total, 157 adolescents consented to be contacted. Each adolescent was then contacted via an initial phone call and followed up with a message (using WhatsApp) in vernacular language detailing the eligibility requirements for a YAB member for Project SAMA and the process of selection. Recruitment materials were designed in simple, jargon-free language and shared in multiple formats (written, verbal, and video) to accommodate different literacy levels and communication preferences.

2.3 How we selected

2.3.1 Application process

All 157 adolescents initially indicated they would apply. Prospective candidates were given 10 days to complete two tasks as a part of the application process.

> Task 1: create a 2–4-minute video sharing their perspective on adolescent mental health as they (as an adolescent) perceived it.
>
> Task 2: "write an essay (500 words) on adolescent mental health."

These tasks were designed to assess their suitability for the YAB by understanding their interest, views, presentation skills and knowledge of mental health. Although these appear demanding tasks, they were suggested by Indian professionals from the Centre for Mental Health Law and Policy (**https://cmhlp.org/**) who have experience in establishing YABs for mental health research. These tasks were considered culturally and youth appropriate, leveraging adolescents' familiarity with digital media and storytelling, allowing them to express their perspectives creatively and authentically. We considered that an essay was a task familiar to them from school and would help us to understand their views on and interest in mental health. The modalities allowed for different communication strengths.

We received application from 90 adolescents. From these, 31 were shortlisted based on external evaluations from two raters with rich experience in the field of child and adolescent mental health. They independently reviewed and rated the applicants video bite and write up, scoring them between 1-10. Evaluators marks were based on the adolescent's:

- Presentation skills
- Confidence while speaking
- Clarity in their communication
- Experience related to mental health
- Clarity in writing
- Content relevance in the writeup

2.3.2 Interview process

The shortlisted 31 applicants then went on to be interviewed by YAB leads. These interviews were conducted online (due to COVID-19) and with the adolescent's consent at the start of the interview, which lasted approximately 10 minutes. The following interview questions were asked:

- to introduce themselves.
- to explain their lived experience
- about their interest and reasons for wanting to join the SAMA project and the YAB

- about their likes and hobbies

At the point of invitation, we reminded applicants of the exclusion criteria (currently living with a serious mental health condition) as if so, YAB involvement may not be suitable for them but that support could be sought for them if they wanted this. No adolescent declared a mental health condition.

Applicants who were successful were those who conveyed strong interest in collaboration, in mental health, and in representing young people. Applicants who were unsuccessful were contacted with an affirming message indicating they were not successful due to the need to represent people from diverse communities.

2.3.3 Final appointments considering diversity

Following interviews, the Yab leads generated a final list of YAB members with a view to appointing as diverse a group of young people as possible. We offered places to 17 members aged between 14 and 18 years all accepted. Originally, we planned to form a single YAB. However, it was decided that two YAB subgroups would be better considering their different locations (rural and urban). Their age range between 14-18 years with the mean age 15.88 years.

3.0 Orientation, training and 'baseline' measures

3.1 'Baseline' measures

To support the chances of YP securing personally meaningful benefits from YAB participation, the lead and deputy lead received training to deliver the Youth Star tool.

- a fee-based, coaching and outcomes assessment tool, where a youth leader / facilitator helps a young person to identify goals for themselves and measure their 'journey' to goal achievement over time.
- Delivered in a one-to-one format, the tool invites adolescents to consider if they feel 'stuck', 'considering', 'having a go', 'working on it' or 'enjoying and achieving' across the six life domains of: interests and activities; hopes and dreams; health and wellbeing; education and work; communication; choice and behaviour.

We felt this would be a usual measure to help us get to know the young person, to help them derive a broader personal benefit from joining our YAB and to understand if YAB membership helped them achieve some of their goals. Our intention was for the YAB leads to administer the tool one-to-one with YAB members at the start, mid-point and end of the YAB.

However, due to logistical and resource challenges, we were unable to deliver this tool as planned.

3.2 Orientation and training for YAB members

An initial orientation meeting was held for YAB members to meet each other and begin to understand the project.

We discussed:

a) **the roles and responsibilities of the YAB members** and began discussions about what they might like from participation. The table here shows examples that were offered to the YAB to stimulate discussion.

YAB Members	YAB Leads
• Attend and participate in meetings<br>• Lead decision making in regard to project problems, content and implementation<br>• Work with the YAB leads to draft the meeting agendas<br>• Facilitate meetings<br>• Keep meeting minutes<br>• Facilitate conversations among each other<br>• Actively voice opinions<br>• Ask if they do not understand something	• Build community<br>• Have consistent, structured meetings<br>• Reflect on meeting feedback<br>• Provide food/ snacks for meetings<br>• Communicate meetings times and locations<br>• Send out reminders about meetings and events<br>• Conduct short educational lessons at the start of meetings based on the topic that is being covered<br>• Keep minutes and feedback often

b) **generating ground rules / code of conduct together.** These can set the foundation for a respectful and productive climate in a YAB. Determining these together at the beginning (and checking in with it when needed) can ensure that the YAB is a safe space. The table here shows examples that were offered to the YAB to stimulate discussion.

1. Respond to the idea, not the person
2. No swearing, name calling, or yelling at other members
3. What happens here stays here
4. Be wary of assumptions
5. Challenge others respectfully
6. Have fun
7. Step up, step back (if you're sharing a lot, step back and let someone else share; if you tend to be quiet, try to step forward more)

c) **how our wider SAMA safeguarding protocol would be applied to the YAB,** emphasising the support available for disclosures of child protection issues, how to disclose and the possible steps that would follow. This was done via discussion of vignettes, e.g.:

"A female adolescent Megha had reported to the facilitator Apurba that one of the workshop participants Ashutosh has been trying to reach out to her through phone (calls and texts). She was feeling uncomfortable about the situation, felt that she could not ask them to stop and asked the facilitators to intervene."

d) **Training the YAB;** Given the lack of existing resources, we designed training for our YAB members involving:

1. **Skill development workshops on effective communication skills**. We asked YAB members ways to think of ways to:
    - create a good online meeting (cameras, using hand functions, being in a quiet space, use of chat function and ensure everyone practices)
    - show you have listened to someone (e.g. by asking a follow up question. *Example activity*: ask people to rate how well the YAB leads listen to each other in a fun mock situation)
    - present their ideas (e.g. making some notes so they are clear what they want to. *Example activity*: ask people to each contribute when they are ready to a discussion about the best weather)
    - convey a different view to someone else (e.g. how to validate different views and feel you can say yours. Challenges in doing this with an adult. *Example* activity: ask two people to disagree in a hyper polite way about the best TV program)
    - work as a team (e.g. acknowledging different skills and personalities; what consensus means and how to be okay with your ideas not being taken forward. *Example activity:* design the perfect day; what 5 items could you agree to all take to a Wanerla picnic spot?
    - showing respect (e.g. for the efforts people make). *Example activity:* list ways that you think this team could show respect to each other.
    - Confidence in speaking. How can we help each other feel more confidence? Example: talk about your favourite subject for 30 seconds

2. **Understanding research and how YABs can influence it.** We provided an overview of:
    - what research is and why it matters, relating to Project SAMA. Example activity: think of other words that mean 'research; (e.g. to find out, to learn, to understand)
    - types of research (e.g., surveys, interviews, interventions) and what was used in Project SAMA, especially the meaning of co-design and feasibility

testing. Example activity: co-design a post-school activity? How would you test if young people liked it?
   - how youth input influences research design and decision-making, including examples from other studies. Example activity: show a basic research outline to find out if adolescents are getting good sleep. How might adolescents help researchers design this study? How might adults go about it the wrong way, or in a way that shows they do not understand young people's lives and concerns?
   - basic ethical principles (privacy, consent, respect for participants). Example activity: vignettes exploring whether a study has secured consent and shown respect to participants

3.3 Setting up meetings

- Seek youth input on the meeting times to maximise attendance and allow the adolescents to be present with minimal stress or inconvenience.
- Common feedback is that the adolescents like regular and frequent meetings.
- Discuss where young people prefer to meet, although this may be restricted by logistical and other parameters.
- Identify how each young person will get to and from the meetings, and any plans for chaperones.

We arranged to meet quarterly for up to two hours each time. One group met on school premises and another at NIMHANS.

4.0 The YAB meetings

4.1 Following a format

A structured format was followed for every meeting, which was important for young people to feel clear about what would be happening each time

(i) *Welcome activity*: a light-hearted, quick check-in activity to give everyone a chance to 'speak into' the space (e.g. favourite ice-cream flavour, if you were a weather today, what would you be?, if you were an animal today, what would you be?; describe a rose and a thorn from your week)

(ii) *Agenda Review*: the topics to be discussed and the goals for the session

(iii) *Activities and Discussion:* focused on a particular need in the project

(iv) *Reflection period*: this can allow YAB members to think back on what has been accomplished, how they feel, and what they may choose to do differently next

time. This will also help guide their agenda-planning for upcoming meetings and identify areas in which they want to improve in terms of group norms/rule enforcement, meeting facilitation, etc.

(v) *Goal Setting:* for the next meeting session, providing clear direction and purpose for the group's ongoing work.

4.2 Recording meetings

This is easiest if there is consent for audio recordings. It is almost impossible for YAB leads to make good notes and facilitate at the same time. Having one person lead the group and another makes notes is possible – which is what we did.

Full minutes were written up and logged securely for our records.

4.3 In-between meetings

YAB leads were in constant WhatsApp communication with the YAB members between meetings, sending friendly messages, reminders and responses.

Feedback was sought after each meeting via open questions (e.g. any thoughts since the meeting? Any feedback for us?)

These were logged by the leads, and any follow-up was reported at the next YAB meeting.

4.4 Example meetings

4.4.1 Creating a shared mission

To build a sense of team and agency, we invited YAB to develop a mission statement. We presented some ideas to stimulate discussion but emphasised the need for their view and creativity. However, this may be difficult to at project start when the YAB members are still discovering about the roles and the project.

Our YAB Members will help Project SAMA by:

- *Providing insight on youth perceptions, interests and needs to ensure SAMA interventions are relevant and meaningful to adolescents.*
- *Advocating for their emotional well-being in school, guiding the project across its three years to ensure a youth-led voice contributes to key decisions.*
- *Providing input into key decisions, reports and events.*
- *Leading some aspects of social media and other communications in the project, focusing on reaching other young people.*

- *Supporting the film-making crew in youth advocacy.*

4.4.2 Exploring safeguarding

We explored the topic of safeguarding in meeting two, once we had established some sense of team. It was important to talk about this early in our work, as the safeguarding commitments related to the YAB as well as to participants in the Project. Example meeting:

Discussion of SAMA Safeguarding Protocol
1. **What does being safe in school mean to you?**<br>Prompt if they find this difficult, e.g. "that bullying is not tolerated", "girls and boys are treated equally", "only positive rewards for good rather than harsh consequences for bad/wrong behavior or opinions",<br><br>2. **What are the steps that you would prefer to be taken when any risk has been reported by an adolescent?** (work in pairs initially)<br>• Explain what risk is: Explain that risk can very and includes: suicide/self-harm, sexual abuse, physical abuse, risky behaviors (like smoking and unprotected sex), bullying, corporal punishment or exploitation.<br>• Explain to the YAB members you are going to go through two examples then you would like to discuss the steps they would prefer to be taken (e.g. did you feel some part of the responses was wrong? Would you like something else to happen when we respond?)<br>• Discuss Vignette 1: *"A female adolescent Megha had reported to the facilitator Apurba that one of the workshop participants Ashutosh has been trying to reach out to her through phone (calls and texts). She was feeling uncomfortable about the situation, felt that she could not ask them to stop and asked the facilitators to intervene."*<br>• Discuss Vignette 2: *"After a SAMA session, an adolescent Uma seemed very distressed during the entire session. She was spoken to later by the facilitator Priyanka. After a while, during the conversation, Uma made some reference of an incident that had happened a few months ago. Priyanka was really concerned and so spent some more time with her and eventually, Uma was able to disclose that she had inappropriately been touched by one of her family members. She spoke more about the incident to Priyanka and said that she had kept this a secret and was sharing this for the first time. Uma was worried about her parents' response to this disclosure and about the conflict it could create in the extended family."*<br>• Explain the POCSO ACT (Protection of Children against Sexual Offenses Act)<br><br>3. **How involved would you want teachers/parents to be in case of disclosure or in managing any risk (in case of low, moderate and high risk) that has been reported?**

<u>Examples of Low Risk</u> = taking screenshots /photos in the middle of a SAMA session or reading a sad example of student stress and feeling down afterwards.<br><u>Examples of Moderate Risk</u> = cyber bullying, inappropriate comments, or use of inappropriate language.<br><u>Examples of High Risk</u> = persistent thoughts of self-harm or suicide or sexual assault.<br>• Pointers to help guide this conversation:<br>▪ Do you think it's important following a risk disclosure that we talk through with the adolescent the steps we as adult researchers may have to take, before we take them?<br>▪ Do you think it's important that the adolescent has a say in who their trusted person is?<br>▪ How do you think we should handle a disclosure when the adolescent says "don't tell me parents" or says "don't tell my teacher" but we feel the adolescent is as risk and we as researchers are concerned for their safety?<br>▪ How can we ensure that adolescents don't think we have broken their trust when we have to involve a teacher or a parent following a disclosure?<br><br>4. **What would be the role of the lay counsellors in the assessment, reporting or management of a potential safeguarding risk?**

4.4.3 Exploring aspects of the intervention

Various aspects of the co-designed intervention were brought for YAB reflection and guidance at different stages. Here is an example of a meeting plan to explore the delivery agent for the proposed whole school program in Project SAMA. These discussions were conducted in a whole group, in pairs or by voting (good for a quick sense of consensus).

**Wellness Coaches training/ classroom integration**
During this meeting we will be discussing with the YAB members all things lay counsellor (we are now calling these 'wellness coaches'). This session will be broken down into the following sections:<br>1. Classroom atmosphere/ dynamic – wellness coach involvement<br>2. Adolescent-wellness coach relationship/ trust building<br>3. Wellness coach-school teacher relationship<br>4. Wellness coach- greater school relationship<br><br>**<u>Section One – Classroom atmosphere/dynamic and the wellness coach's involvement and responsibility.</u>**<br>What kind of environment do you think the wellness coach needs to create to ensure the SAMA sessions are best delivered to the adolescents? These could be similar to

the roles and responsibilities that you created previously for me as the YAB coordinator, or they may be similar the rules/ code of conduct that you set for the YAB group.

Examples:

- Fun, friendly atmosphere
- Private and confidential sessions
- More informal than a typical lesson this might mean a different seating format e.g. in a circle or different positions
- You get to sit next to your friends
- This is a safe space with no judgment or consequences

Key question: Do you think a teacher should be present during the SAMA sessions to help with class management or would this make to adolescents less likely to be open and involved in the sessions? (e.g. would they feel judged and more scared to open up)

Instead, do you think the adolescents would prefer another person (member of the SAMA research team instead who doesn't know them at school like myself?) Would they find this more off putting or comforting?

Section Two - Adolescent-wellness coach relationship/ trust building

- Do you think its important for the wellness coach to be identifiable as different to a teacher for adolescents? If so, why? And how could this be done?
- How could the wellness coach build trust with the adolescents?
- Do you think adolescents need to know how/ where to locate or contact the lay counsellor outside of SAMA sessions? If you think this is import why so?
- What questions do you think the adolescents will have about the SAMA wellness coaches?

Section Three - Wellness coach-school teacher relationship

- How do you think the wellness coaches should interact with the teachers at the school?
- If there is a school councillor already present at the school how should the SAMA wellness coach interact with them?
- What questions do you think teachers will have about the SAMA wellness coaches?

Section Four - Wellness coach- greater school relationship

- Do you think the wider school members (e.g. other adolescents, teachers and school staff) need to know who the SAMA wellness coach is? And that they are different to a teacher? If so how could we do this?
- What questions do you think other school members will have about the SAMA wellness coaches?

4.4.4 Exploring adolescents' lives

As new insights or uncertainties arise in a project, it can be helpful to ask the YAB to shed more light on these.

From our co-production sessions with another group of young people, it became apparent that they did not feel their parents supported them or were approachable to discuss their problems with (including problems of their well-being), and similarly that they parents did not know how best to communicate with them. We brought this for further exploration with the YAB as this was important to developing our parent workshops.

Closing the Communication Gap
1. Are the YAB members surprised to hear what other adolescents have conveyed about a communication gap with parents?<br>2. Might this be more difficult for some young people than other - why might this be?<br>3. How does it feel to be compared to siblings or other young people by parents– might the effect of this be different for different young people?<br>4. How could your parents make themselves more approachable to their child? So they feel more able to talk to them about your problems and well-being?<br>5. How would you like your parents to start a conversation with you about your well-being?   Create top tips for parents / conversation starters created (aim for at least 5 conversation starters to be created by the YAB)<br>6. How could your parents better support you and specifically your emotional wellbeing? What might this practically look like?

4.4.5 Exploring how to optimise data collection

We were keen to ensure that our planned measures, and methods of administration, made sense to adolescents. In one meeting we explained the importance of standardised measures, and some of our worries about how administering them. We invited the YAB to be solution-finders with us. Example questions:

- How can we make it clear to adolescents that these measures are not a test, and their answers will be kept confidential?
- Is it confusing how to answer the questions e.g., circle/tick/changing time frames? How can we make this clear to adolescents?
- Is any of the language used in the measures confusing? Are there any words you don't understand ? if so, what word could we use instead? Or could you create a definition of the word(s)?
- How can we make it clear that the time periods the measures are referring to alter for each measure? Is this confusing for adolescents? How can we make this clearer?

4.4.6 Exploring project outputs and how to optimise them

In Project SAMA, young people in the intervention schools created films. We brought these to the YAB to explore their response to them and creative ideas for optimising their impact. An example meeting structure:

**Show SAMA films + group discussion/questions**
• What was the part of the films you liked the most? / What about the stories did you like the most?<br>• If you had to make changes like add something or make something a little different, what would you do?<br>• Did watching these films add to or change your understanding of Project SAMA? Or your understanding of well-being?<br>• If you could make a film like this, what would your film be about?<br><br><u>Credibility of the SAMA stories</u><br>Ask<br>• if they have experienced or witnessed similar situations to those in the SAMA films.<br>• if they feel these situations are relevant to the life of an Indian school going adolescent<br>• how they would like/prefer to seek help in their schools? Discuss barriers and facilitators of help seeking in their school.<br><br><u>Teacher's views questions:</u><br>Discuss with the adolescents the teachers and head teachers' views of Project SAMA they were mentioned in the SAMA films. Ask the adolescents if this is what they expected their teachers and head teachers to think? Ask the adolescents why the head teacher and teacher liked SAMA? Discuss with the adolescents the other benefits they believe SAMA would bring to their school.

5.0 Concluding the YAB

It is important to think about preparing the YAB members for the end of the project. It can help to plan the ending together and how achievements can be marked.

We discussed with YAB members about their preferences for our final session, and they asked for time to discuss skills to manage their daily difficulties. Two experienced mental health researchers facilitated life skills sessions with the YAB, focusing on managing emotions, decision making, interpersonal relationships and stress management.

To formally and publically recognise and celebrate the work of the YAB in Project SAMA, all YAB members were invited to NIMHANS; we felt it was important to invite them to the Institute rather than hold a school-based event to foster their sense of place and belonging in professional and civic life.

The final celebration event included summaries of the work and impact of the YAB and expressions of gratitude; all received a small memento and were thanked formally for their contribution.

We also sought feedback via an anonymous link asking the YAB members to feed back on any aspect of their time on the YAB, what benefits they may have gained, if any.
